# Predictors and health outcomes of tobacco smoking among the population of Gaza: A large-scale study

**DOI:** 10.21203/rs.3.rs-4195976/v1

**Published:** 2024-04-22

**Authors:** Ali Ismail, Layal Hamdar, Hiba Dirawi, Mohamad Kanso, Islam Salem, Hani Tamim, Ziyad Mahfoud

**Affiliations:** American University of Beirut Medical Center; American University of Beirut Medical Center; American University of Beirut Medical Center; American University of Beirut Medical Center; American University of Beirut; American University of Beirut Medical Center; Weill Cornell Medicine

**Keywords:** Smoking, tobacco, cigarette, shisha, NCD, Gaza

## Abstract

Tobacco smoking, a significant public health concern globally, is associated with a rise in noncommunicable diseases and preventable deaths, with pronounced impacts in conflict zones like Gaza. A study in Gaza focused on individuals over 40 years of age, aiming to identify predictors of tobacco use and its links to diseases like coronary artery disease, chronic lung disease, and stroke. The research, based on the Gaza NCD study data with 4576 participants and a 96.6% response rate, found an overall tobacco smoking prevalence of 19.4%, with higher rates among men. Adjusting for various factors, the study revealed significant associations between cigarette smoking in men and adverse health outcomes, such as coronary artery disease and chronic lung disease. However, after adjusting for independent variables, shisha smoking in men showed no association with these health outcomes. In conflict-affected regions like Gaza, this large-scale study sheds light on predictors of cigarette and shisha smoking and their impact on health outcomes, offering valuable insights for researchers, public health officials, healthcare professionals, and policymakers. The findings aid in predicting smoking prevalence, addressing current health challenges, and mitigating potential health and financial burdens associated with tobacco use in conflict zones.

## Background

Tobacco smoking is considered a global public health concern that is associated with significant morbidity and premature mortality.^1,2^ The number of smokers worldwide increased to 1.1 billion in 2019, with tobacco smoking leading to 7.7 million deaths.^3^ The prevalence of tobacco smoking, including cigarette and shisha smoking, is particularly increasing in the Middle East and has been alarming in some Arab countries.^4^ Tobacco smoking remains one of the leading causes of preventable deaths and is a primary risk factor for the increase in noncommunicable diseases (NCDs) and tobacco-specific morbidities affecting mainly the cardiovascular and respiratory systems.^5^ In fact, the adverse health effects of smoking include but are not limited to coronary artery disease, chronic obstructive pulmonary disease and lung cancer.^6^ The burden of treating these smoking-related diseases contributes to a substantial economic strain on the healthcare system. This is evident in the increased costs for medical treatments, hospitalizations, medications, and the necessary care for addressing these chronic medical conditions.^7^

Smoking is also driven by various socioeconomic and psychological factors. In fact, tobacco smoking has been found to be more prevalent among those with lower socioeconomic status (SES).^8^ The high prevalence of smoking among disadvantaged groups is suggested to be influenced by low social support, low levels of awareness and education, reduced motivation to quit, a stronger addiction to tobacco use, lack of self-efficacy, and the impact of tobacco industry marketing, particularly due to low tobacco prices.^9^

Tobacco smoking in the Arab region has also been a significant concern for public health officials, health professionals, and policy makers.^4^ In fact, the prevalence of tobacco smoking has been particularly high in Arab men compared to women.^10^ However, there has been very little global research addressing the use of tobacco and nicotine dependence among civilians in conflict-affected areas, despite key vulnerabilities such as the presence of various socioeconomic stressors. Most studies have addressed this topic within adolescent and young adult populations.

Indeed, a study performed in the Republic of Georgia revealed that nicotine dependence was significantly associated with multiple mental health disorders in older conflict-affected men.^11^ Moreover, the Palestinian Central Bureau of Statistics (PCBS), in its preliminary results of the “Smoking and Tobacco Consumption Survey, 2021”, reported an obvious increase in the prevalence of smoking among individuals aged 18 and above in the West Bank.^12^

As such, many efforts have been made to address the predictors of smoking, given its association with the challenging burden of disease, disability, and death. Among these predictors, we mention socioeconomic status, cultural norms, age, and gender.

Past studies have examined the prevalence of smoking and awareness of smoking-associated health risks among young people in Gaza, specifically focusing on university students. These studies were relatively small in size.^13,14^

The objectives of this study were to identify the predictors of tobacco smoking among a large representative sample of individuals who were at least 40 years of age and who were living in Gaza and to assess the associations between tobacco smoking and noncommunicable diseases, mainly coronary artery disease (CAD), chronic lung disease (CLD) and stroke.

## Methods

### Study Design

This was a secondary data analysis of the Gaza NCD study database. The 2020 cross-sectional study collected data on a representative sample of 4576 individuals aged 40 years and above selected from 2493 households across Gaza’s five governorates through an interviewer-administered household survey. Prior to data collection, interviewers visited each household for the study and obtained verbal informed consent. In each household, one eligible male and one eligible female were interviewed and included in this study. The aim of collecting this dataset was to assess and identify potential solutions for noncommunicable diseases (NCDs) in a densely populated area affected by prolonged armed conflict, such as Gaza.^15^ The study received approval from the Imperial College Research Ethics Committee (reference 20IC5733), the American University of Beirut Institutional Review Board, and the Gaza Helsinki Committee (reference PHRC/HC/483/19). All methods conducted for the manuscript adhered to applicable guidelines and regulations.

Current cigarette smoking status was defined according to the World Health Organization (WHO) definition. This definition encompasses individuals who have smoked 100 or more cigarettes over their lifetime and currently engage in daily smoking or smoking on some days. Current shisha smoking was defined as smoking Shisha either daily or on some days.

Our primary objective was to identify predictors of both cigarette and shisha smoke exposure. The selection of these predictors was based on known risk factors for smoking documented in the literature, as well as the availability of relevant data within the Gaza dataset.

Age in years was categorized into four categories: 40–50, 50–60, 60–70, and > 70 years.

Educational levels were classified into four groups: illiterate, basic education (able to read and write, elementary, preparatory), intermediate education (secondary, associate diploma, bachelor’s degree), and higher education (higher diploma, master’s degree, PhD).

We defined physical activity as the initiation or increase in physical activity within the past year and categorized it as “yes” or “no.”

Body mass index (BMI) was initially classified into six categories: underweight, normal, overweight, obese class I, obese class II, and obese class

III. Subsequently. We recategorized the data into three categories: normal/underweight, overweight, and obese.

Marital status was categorized into two groups: married and unmarried.

Other predictors, such as having worked during the past 30 days, having health insurance and receiving cash assistance, were all dichotomized as yes/no.

The presence of coronary artery disease was assessed if participants reported a history of heart attack or angina. It was dichotomized as yes/no.

Chronic lung disease was assessed by determining whether participants had a history of chronic obstructive pulmonary disease (COPD), asthma, or respiratory allergies and was dichotomized as yes/no.

Stroke was assessed by determining whether participants had a history of stroke and was dichotomized as yes/no.

Independent risk factors for the aforementioned health outcomes included smoking cigarettes and shisha, which are the same independent variables for the primary outcome, as well as hypercholesterolemia and hypertension.

Hypercholesterolemia and hypertension were assessed by determining if participants had previously received a diagnosis of high cholesterol or high blood pressure (or systolic blood pressure > 140 mmHg or diastolic blood pressure > 90 mmHg), and they were dichotomized as yes/no.

### Statistical analysis

Demographics and other variables of the study were summarized using frequency distributions. The prevalence of cigarette and shisha smoking were computed along with their 95% confidence intervals. Univariable and multivariable logistic regressions were used to identify the predictors of cigarette smoking and shisha smoking. Unadjusted and adjusted odds ratios are presented along with their 95% confidence intervals. Hosmer and Lemeshow tests were used to assess the goodness-of-fit of the models, and the predictive power of the models was assessed using receiver operating characteristic (ROC) curves. Similar analyses were performed to assess the potential association between tobacco smoking and noncommunicable diseases. IBM-SPSS (version 29, Armonk, NY, USA) was used for the data analysis. Statistical significance was set at the 5% level.

## Results

A total of 4,576 participants were included in the cross-sectional study, resulting in a response rate of 96.6%. Of the participants, 46% were males, and 38% were above 60 years of age. For more details, Abu Hamad *et al*. provided a description of this sample.^15^

**Figure 1:**
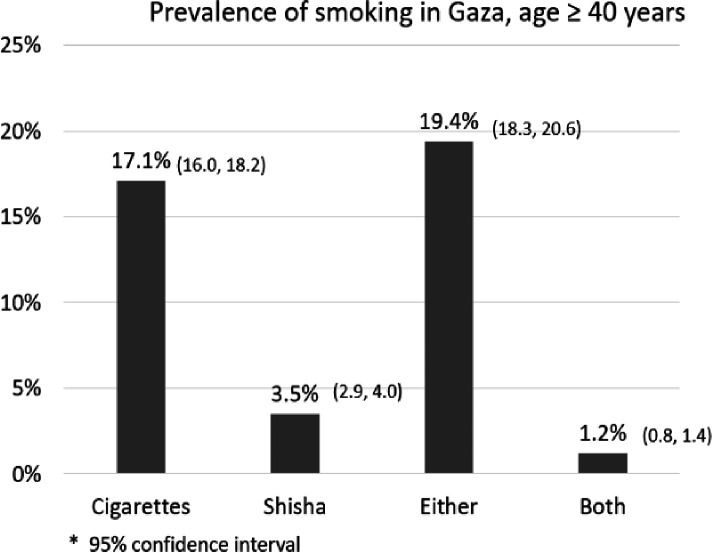
Prevalence of cigarette smoking, shisha smoking, and use of both among individuals aged 40 years and older in the Gaza population.

**Figure 2:**
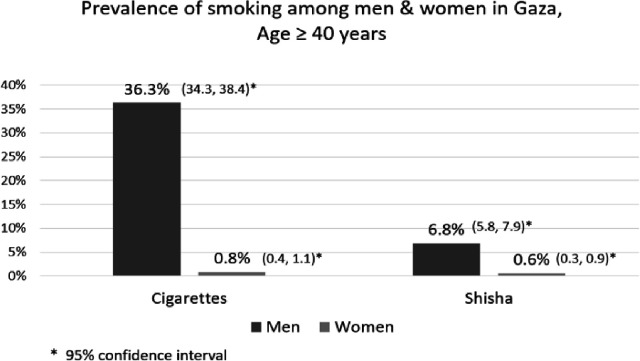
Prevalence of smoking cigarettes and shisha among men and women in Gaza, aged ≥40 years.

The prevalence of current cigarette smoking was 17.1% (95% CI: 16.0%–18.2%), and for shisha smoking, it was 3.5% (95% CI: 2.9%–4.0%). Among the participants, 783 individuals (19.4%) were identified as current cigarette or shisha smokers, with only 1.2% reported as users of both (refer to Fig. 1), but there was a marked sex difference. Given the higher prevalence rates among men (36.6% for cigarettes and 6.8% for shisha) than among women (0.8% for cigarettes and 0.6% for shisha), our analysis focused exclusively on male participants (see Fig. 2). The unadjusted and adjusted odds ratios of cigarette and shisha smoking are shown in Tables 1 and 2.

For men, at the bivariate level, younger age, nonobesity status, and lack of health insurance were significantly associated with increased odds of current cigarette smoking. Additionally, being younger, being working during the past 30 days, and receiving no cash assistance were associated with greater odds of current shisha smoking.

Those between 40 and 50 years of age were the most likely to smoke cigarettes (48.7%), whereas individuals above 70 years of age were the least likely (17.3%) to smoke cigarettes. A similar pattern was observed for shisha smoking, with 12.2% between 40 and 50 years of age and 1.2% among those above 70 years of age, both showing statistical significance (p value 0.001). Additionally, individuals with a lower/normal BMI (59.5%), who had a basic education (42.1%), and who lacked health insurance (43.7%) were more likely to smoke cigarettes (Table 1).

**Figure 3:**
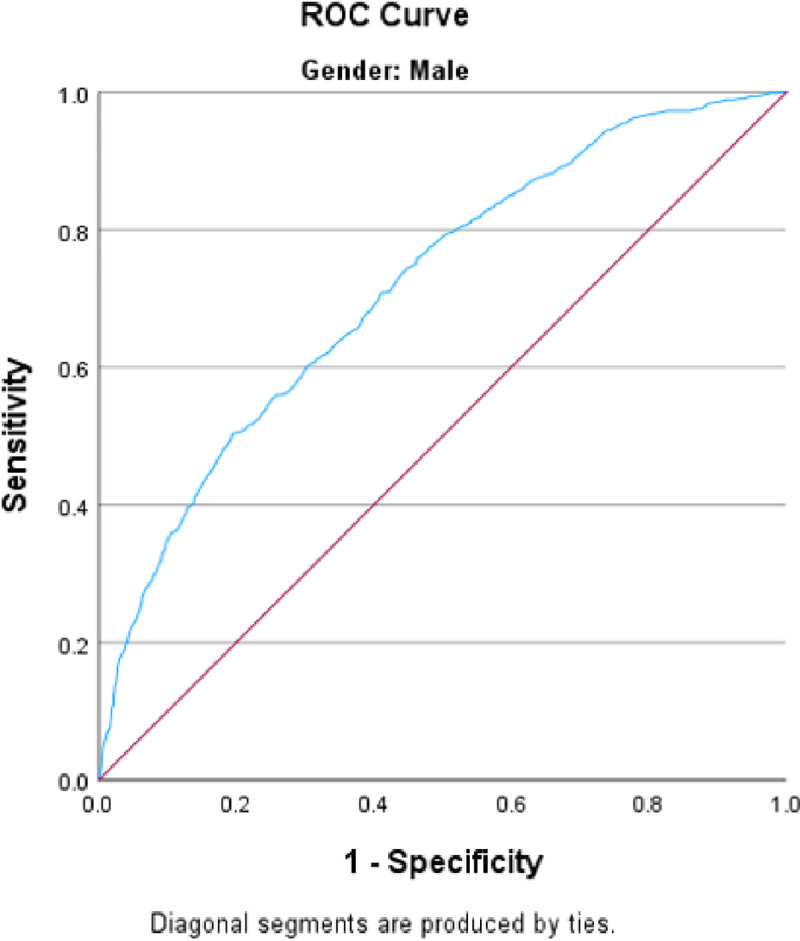
ROC curve of the cigarette smoking model for men. Area under the curve = 0.717

**Figure 4:**
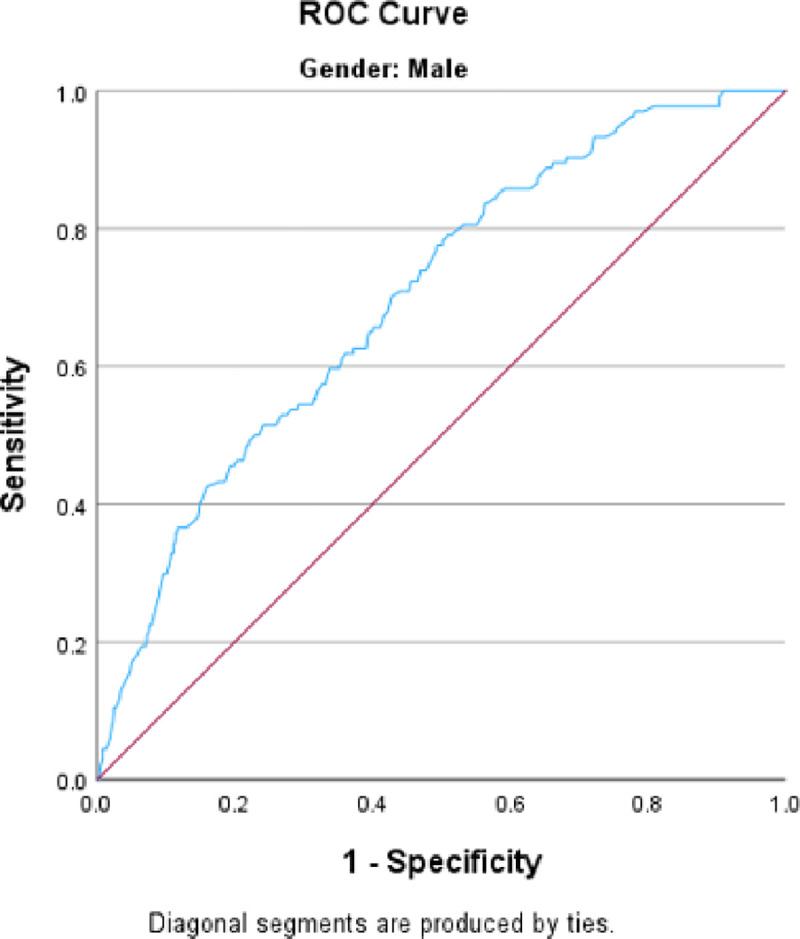
ROC curve for men in the Shisha smoking model. Area under the curve = 0.699

Table 1 and Table 2 show the associations between the independent variables and the risk of smoking cigarettes and smoking Shisha rice in men. We calculated the adjusted odds ratios while considering adjustments for age, BMI, education, increase in physical activity over the past year, health insurance, marital status, cash assistance, and working status during the previous 30 days. Multivariate analysis of cigarette smoking (Table 1) revealed that older individuals were less likely to smoke [OR = 0.161, 95% CI (0.11, 0.24)]. Individuals with intermediate education, in contrast to those who are illiterate, exhibit a lower likelihood of smoking [OR = 0.528, 95% CI (0.31, 0.90)]. Increased physical activity over the past year was associated with a reduced likelihood of smoking [OR = 0.762, 95% CI (0.62, 0.93)]. Moreover, individuals with a higher body mass index had a lower chance of smoking [OR = 0.762, 95% CI (0.62, 0.93)], while those with health insurance were less inclined to smoke [OR = 0.764, 95% CI (0.59, 0.99)]. Having worked during the previous 30 days, having cash assistance and marital status were not associated with cigarette smoking. The goodness-of-fit of the model using the Hosmer‒Lemeshow test showed a good fit (p value of 0.394) and the ability to predict smokers and nonsmokers correctly in 71.7% of the patients (Fig. 3). In the Shisha model, after adjusting for all the variables in the model (Table 2), only a few variables showed a significant association. Older men [OR = 0.084, 95% CI (0.03, 0.28)] and those receiving cash assistance [OR = 0.566, 95% CI (0.37, 0.86)] were less likely to smoke shisha. Additionally, our findings revealed no association between smoking shisha rice and variables such as BMI, education, increase in physical activity during the past year, having health insurance, marital status, or working status during the previous 30 days. The goodness-of-fit of the model using the Hosmer‒Lemeshow test showed a good fit (p value of 0.269) and the ability to predict smokers and nonsmokers correctly in 69.9% of the patients (Fig. 4).

**Figure 5:**
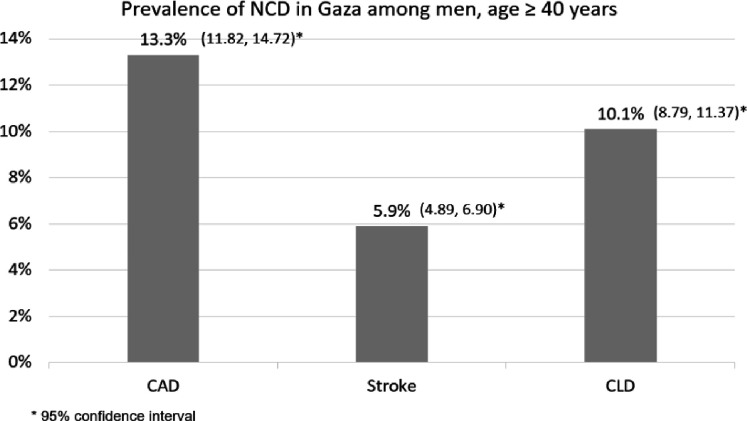
Prevalence of noncommunicable diseases associated with smoking in men

Among men, the prevalence of NCDs was 13.3% for CAD, 5.9% for stroke, and 10.1% for CLD (Fig. 5). Three separate models for men were created, one for each of these conditions. We calculated the adjusted odds ratios for variables such as age, education, increase in physical activity during the past year, BMI, marital status, work during the past month, health insurance, cash assistance, hypercholesterolemia, hypertension, cigarette smoking, and shisha smoking. The goodness-of-fit of the CAD, CLD, and stroke models was assessed using the Hosmer–Lemeshow test, which indicated good fit, with p values of 0.134, 0.523, and 0.591, respectively. Our analysis revealed that cigarette smoking is significantly associated with a history of CAD and CLD. However, no significant association was found between smoking cigarettes and stroke incidence. Moreover, there was no association between smoking shisha rice and any of the three aforementioned health outcomes (Table 3).

## Discussion

This study identified significant predictors of tobacco smoking (including cigarettes and shisha) in the Gaza Strip. This study with a representative sample aimed to assess the burden of smoking among adults aged above 40 years in Gaza.

In the context of cigarette smoking, a greater likelihood of smoking was associated with being male. Historically, smoking has been more socially accepted among men than among women. Furthermore, findings from neuroimaging data indicate that smoking triggers reward pathways in men more than in women.^17^ Additionally, advertising agencies have traditionally targeted male consumers using role models such as actors and athletes. Given the near absence of smoking among women in the population of Gaza, we focused our analysis exclusively on men. Smoking is nearly nonexistent among women in Gaza, and if it does occur, it is not openly reported due to societal taboos. Women might also be more aware of the negative impact of smoking, especially its impact on pregnancy.^18^

In our study, smoking was also found to be less prevalent among older adults. This can be explained by the fact that older individuals might have quit smoking due to health conditions that make it inadvisable or due to its negative impact on their overall wellbeing. Another plausible explanation is that individuals aged 70 years and above are more likely to be healthy and are initially nonsmokers. ^19^

Our study also revealed an association between being physically active in the past year and reduced cigarette smoking. Individuals who lead a healthy lifestyle by exercising are more likely to be smoke-free. Relying on physical activity is an excellent stress relief mechanism that can be adopted rather than resorting to tobacco smoking.^20^ Recent studies have shown that physical activity is an effective mechanism used in many smoking cessation programs.^21^

Furthermore, our study revealed a negative correlation between intermediate education and cigarette smoking. Educated individuals are better equipped to understand the detrimental effects of smoking and may have a higher socioeconomic status, granting them improved access to healthcare services and the means to participate in smoking cessation programs. Conversely, individuals with intermediate education may face limitations in resources and healthcare access compared to their higher-educated counterparts, potentially resulting in lower smoking rates among this group.^8,22^

Regarding body mass index (BMI), individuals who were overweight or obese had a lower likelihood of being smokers. Smoking has the potential to curb appetite and increase metabolism, leading to smokers often having a reduced BMI. Conversely, individuals with a higher BMI are at increased risk of developing several noncommunicable diseases.^23^

Having health insurance was associated with a lower risk of smoking. Health insurance is a marker of higher socioeconomic status in Gaza. People with health insurance generally have better access to healthcare services. Furthermore, smokers are less likely to purchase insurance deals, which contradicts theoretical expectations given the serious complications that can arise from smoking.^24^

In our study, we found that working in the past 30 days, having cash assistance or being married were not associated with cigarette smoking. Moreover, being elderly or receiving cash assistance was negatively associated with smoking.^14,15^

In relation to the study’s secondary outcomes in men, these data are limited by the fact that they are self-reported and elicited at the same time as the smoking questionnaire. Therefore, they are looking for associations, and no causal implications can be drawn.

Cigarette smoking was found to be significantly associated with a history of CAD and CLD. Conversely, no association was found between smoking shisha rice and a history of these noncommunicable diseases. In fact, studies have shown that the number of cigarettes smoked is associated with the number of damaged vessels and the severity of CAD.^24^ Moreover, according to the Global Initiative for Chronic Obstructive Lung Disease (GOLD) guidelines, approximately 50% of smokers will eventually develop COPD.^25^ In our study, no significant association was noted between cigarette smoking and the risk of developing stroke. Indeed, it was demonstrated in the literature that up to one-quarter of all strokes are directly attributed to cigarette smoking. No association was noted between shisha smoking and the risk of developing noncommunicable diseases such as CAD, CLD and stroke. This might be related to the small population size of people who smoke shisha, which may negatively impact the study’s power to detect a significant difference.

### Limitations/Strengths

The data were collected three years prior to the beginning of the Gaza War on October 7, 2023. The insights derived from these data may remain pertinent for the population and could offer valuable understanding of the risk factors for noncommunicable diseases associated with smoking, particularly in a population currently contending with a healthcare system that has completely collapsed. Despite war-related destruction, preconflicting data remain a vital resource for informing public health strategies, shaping policies, and guiding long-term health planning and interventions during the reconstruction of Gaza’s healthcare system.

The current study provides valuable insights into the predictors of cigarette and shisha smoking in the Gaza population. Although this is one of the largest reports on the prevalence of smoking in Gaza, there are several limitations to consider. First, the study’s cross-sectional design prevents us from establishing temporality and determining whether subjects were exposed to smoking before or after contracting diseases.

Another limitation of the study is that all variables, including smoking and reporting NCDs, relied on self-reported data. This introduces the possibility of overestimation or underestimation in some cases, potentially affecting the accuracy of the findings. Additionally, due to the low prevalence of shisha rice, the statistical models employed may lack power or may not fit the data well.

Moreover, as this study was not primarily designed to investigate smoking as the main outcome, there may be additional predictors of smoking that were not included in the analysis, such as family history of smoking, peer pressure, advertising and media.

It is important to acknowledge these limitations, as they provide context for the findings and emphasize the need for further research to overcome these challenges and gain a more comprehensive understanding of smoking behavior in the Gaza population.

The current study on the predictors of cigarette and shisha smoking in the Gaza population is a significant contribution to the literature. This study not only provides important information on smoking patterns in the Gaza population but also sheds light on the risk of developing noncommunicable diseases such as cardiovascular diseases and chronic lung disease. Several key strengths of the study can be highlighted. First, the study boasts a reasonably large sample size, ensuring that the findings are based on a substantial number of participants. This approach enhances the statistical power and reliability of the results. A notable strength is the representative nature of the sample, which ensures that the prevalence rates of smoking and associated risk factors accurately reflect the population of interest. Another strength of the study lies in its ability to assess multiple outcomes and predictors. By examining the relationship between smoking and various diseases, this study provides a comprehensive understanding of the risks associated with smoking.

## Conclusion

In conclusion, our study revealed several important predictors of smoking habits. Notably, we found a positive correlation between cigarette smoking and coronary artery disease (CAD) and chronic lung disease, while no significant correlation with stroke was identified. This study represents a valuable asset for researchers, policymakers, and healthcare professionals engaged in efforts to prevent and control smoking among the population in Gaza. This information will be essential for informing the reconstruction of the healthcare system in Gaza following the war.

## Figures and Tables

**Table 1:** Cigarette smoking model in men

Men	Nonsmoker (Cigarettes) N(%)	Cigarette Smoker N(%)	Unadjusted OR (95% CI)	P value	Adjusted OR (95%CI)	p value

Age						

40–50	215 (51.3)	204 (48.7)	1		1	

50–60	452 (57.9)	329 (42.1)	0.767 (0.60, 0.97)	0.029	0.750 (0.58, 0.97)	0.03

60–70	389 (69.3)	172 (30.7)	0.466 (0.36, 0.61)	<0.001	0.448 (0.33, 0.60)	<.001

>70	283 (82.7)	59 (17.3)	0.220 (0.16, 0.31)	<0.001	0.161 (0.11,0.24)	<.001

Education						

Illiterate	55 (64.7)	30 (35.3)	1		1	

Basic education	610 (57.5)	451(42.5)	1.355 (0.86, 2.15)	0.196	0.911 (0.54, 1.54)	0.727

Intermediate education	638 (71.1)	259 (28.9)	0.744 (0.466, 1.188)	0.216	0.528 (0.31,0.90)	0.020

Higher education	367 (60.0)	24 (40.0)	1.222 (0.618, 2.417)	0.564	1.098 (0.51,2.38)	0.812

Start/do more physical activity in past year	715 (62.8)	423 (37.2)	1	0.384	1	0.008
	624 (64.7)	341 (35.3)	0.924 (0.77, 1.10)		0.762 (0.62, 0.93)	
No						
Yes						

BMI						

Normal/Underweight	181 (40.5)	266 (59.5)	1		1	

Overweight	520 (64.8)	282 (35.2)	0.369 (0.29, 0.47)	<0.001	0.324 (0.25, 0.42)	<.001

Obese	584 (74.8)	197 (25.2)	0.230 (0.18, 0.29)	<0.001	0.199 (0.15, 0.26)	<.001

Marital status	25 (78.1)	7 (21.9)	1	0.093	1	0.405
Unmarried	1314 (63.4)	757 (36.6)	2.058 (0.89, 4.78)		1.491 (0.58, 3.81)	
Married						

Did you work during the past 30 days?	925 (65.0)	499 (35.0)	1	0.083	1	0.428
No	414 (61.13)	264 (38.9)	1.182 (0.98, 1.43)		0.909 (0.72, 1.15)	
Yes						

Health insurance	193 (56.3)	150 (43.78)	1	0.002	1	0.039
No	1146 (65.2)	612 (34.8)	0.687 (0.54, 0.87)		0.764 (0.59, 0.99)	
Yes						

Cash assistance	748 (65.2)	400 (34.8)	1	0.12	1	0.068
No	591 (61.9)	364 (38.1)	1.152 (0.96, 1.38)		0.818 (0.66, 1.02)	
Yes						

**Table 2:** Shisha smoking model in men.

Men	Nonsmoker (Shisha) N(%)	Shisha Smoker N(%)	Unadjusted OR (95% CI)	P value	Adjusted OR (95%CI)	p value

Age						

40–50	368 (87.8)	51 (12.2)	1		1	

50–60	716 (91.7)	65 (8.3)	0.655 (0.45, 0.97)	<0.032	0.738 (0.49, 1.11)	0.148

60–70	537 (95.7)	24 (4.3)	0.322 (0.20, 0.53)	<0.001	0.412 (0.24, 0.71)	0.002

>70	338 (98.8)	4 (1.2)	0.085 (0.03, 0.24)	<0.001	0.084 (0.03, 0.28)	<.001

Education						

Illiterate	81 (95.3)	4 (4.7)	1		1	

Basic education	998 (94.1)	63 (5.9)	1.278 (0.45, 3.60)	0.642	0.991 (0.30, 3.31)	0.988

Intermediate education	827 (92.2)	70 (7.8)	1.714 (0.61,4.82)	0.307	1.152 (0.34, 3.87)	0.819

Higher education	53 (88.3)	7 (11.7)	2.675 (0.75, 9.58)	0.131	1.689 (0.40, 7.18)	0.478

Start/do more physical activity in past year	1061 (93.2)	77 (6.8)	1	0.873	1	0.372
	898 (93.1)	67 (6.9)	1.028 (0.73, 5.11)		0.848 (0.59, 1.22)	
No						
Yes						

BMI						

Normal/Underweight	425 (95.1)	22 (4.9)	1		1	

Overweight	743 (92.6)	59 (7.4)	1.534 (0.93, 2.54)	0.096	1.340 (0.80, 2.24)	0.266

Obese	728 (93.2)	53 (6.8)	1.406 (0.84, 2.35)	0.191	1.277 (0.76, 2.16)	0.361

Marital status	30 (93.8)	2 (6.3)	1	0.893	1	0.494
Unmarried	1929 (93.1)	142(6.9)	1.104 (0.26, 4.667)		0.59 (0.13, 2.67)	
Married						

Did you work during the past 30 days?	1354 (95.1)	70 (4.9)	1	0.001	1	0.207
No	604 (89.1)	74 (10.9)	2.370 (1.69, 3.33)		1.296 (0.87, 1.94)	
Yes						

Health insurance	314 (91.5)	29 (8.5)	1	0.201	1	0.461
No	1643 (93.5)	115 (6.5)	0.758 (0.50, 1.16)		0.845 (0.54, 1.32)	
Yes						

Cash assistance	1047 (91.2)	101 (8.8)	1	<0.001	1	0.008
No	912 (95.5)	43 (4.5)	0.489 (0.34, 0.71)		0.566 (0.37, 0.86)	
Yes						

**Table 3:** Association between noncommunicable diseases (CAD, CLD and stroke) and smoking in men.

	Cigarette Smoking/No						Shisha Smoking/No							
Model	Unadj. odds ratio	CI 95%	P-value	Adj. odds ratio	CI 95%	P-value	Unadj. odds ratio	CI 95%	P-value	Adj. odds ratio	CI 95%	P-value	Hosmer & Lemeshow test	Area under the curve
**CAD**	1.03	0.79–1.34	0.826	1.67	1.22–2.29	0.001	0.576	0.32–1.06	0.074	0.75	0.39–1.44	0.38	0.134	0.785
**CLD**	1.55	1.16–2.06	0.003	1.68	1.21–2.33	0.002	0.8	0.44–1.36	1.47	0.96	0.50–1.86	0.9	0.523	0.657
**Stroke**	0.646	0.43–0.97	0.96	0.82	0.52–1.32	0.415	0.438	0.16–1.20	0.109	0.667	0.23–1.91	0.451	0.591	0.769

We adjusted for age, education, physical activity, BMI, marital status, work during the past month, health insurance, cash assistance, hypercholesterolemia, hypertension, cigarette smoking, and shisha smoking in each of the 3 models.

## Data Availability

The data supporting the findings of this study are available upon request from the Gaza NCD study dataset, in accordance with data sharing policies established by the UK’s Department for International Development (DFID), the Medical Research Council (MRC), the Economic and Social Research Council (ESRC), and Welcome Trust’s Health Systems Research Initiative (HSRI) (Grant number: MR/S012877/1).
